# A unique coincidence: Concurrent oncocytic thyroid Carcinoma and tenosynovial giant cell tumor: A case report

**DOI:** 10.22038/aojnmb.2026.87172.1625

**Published:** 2026

**Authors:** Fatimah AbuAlJaaz, Mahd Foqhaa, Mohammed abduljabbar Abed, Ahmed Saad Abdlkadir, Akram Al-Ibraheem

**Affiliations:** 1 Nuclear Medicine Department, King Hussein Cancer Center (KHCC), Amman, Jordan; 2 Radiology Department, King Hussein Cancer Center (KHCC), Amman, Jordan

**Keywords:** Oncocytic thyroid carcinoma, Tenosynovial giant cell tumor, Whole body iodine scan, FDG PET/CT

## Abstract

Oncocytic thyroid carcinoma (OTC) and tenosynovial giant cell tumor (TGCT) are rare neoplasms with distinct biological behavior, and their coexistence has not been previously well documented. We report a rare case of concurrent recurrent OTC and diffuse-type TGCT (TGCT-D) in a 45-year-old man who presented with a rapidly enlarging soft-tissue mass involving the left ankle and foot. ^18^F-fluorodeoxyglucose positron emission tomography/computed tomography (FDG PET/CT) demonstrated intensely FDG-avid lesions in the thyroid bed consistent with recurrent disease, as well as a hypermetabolic infiltrative musculoskeletal lesion in the left ankle and foot, initially suspected to represent metastatic spread. However, histopathological examination of the ankle lesion established the diagnosis of TGCT-D. This case illustrates an important diagnostic pitfall in oncology imaging: FDG-avid musculoskeletal lesions in patients with known malignancy do not necessarily represent metastases. Careful integration of clinical information, cross-sectional imaging, particularly MRI, and histopathological confirmation is essential to avoid misdiagnosis and to ensure appropriate management.

## Introduction

 To our knowledge, this is the first documented case of the co-occurrence of OTC and TGCT-D. OTC, also referred to as oxyphilic carcinoma and previously known as Hürthle cell tumor, is a rare variant of differentiated thyroid cancer. It account for approximately 3% to 5% of all thyroid cancers, making it considerably less common than other subtypes (1, 2). Histologically, it does not exhibit the distinctive nuclear characteristics of papillary thyroid cancers (PTC) or the features associated with high-grade malignancies (1). 

 Thyroid cancer is twice as common in women as in men; however, men tend to have a worse prognosis. OTC is rare and is associated with high likelihood of recurrence, metastasis, and a poor prognosis (1, 3). Compared with other types of thyroid cancer, OTC has a higher propensity for metastasis to regional lymph nodes and distant organs, including lungs, liver, and bones (2). Recent studies have highlighted the role of radioactive iodine (RAI) therapy in management and improving overall survival particularly patients without extensive metastatic disease. Additionally, FDG-PET scan plays a decisive role in diagnosis, assessing disease extent, and treatment response. However, clinicians should remain aware of the potential for false-positive results, therefore clinical correlation always essential (4).

 TGCT is a rare, locally aggressive mesenchymal tumor including a group of typically benign, proliferative, and inflammatory conditions that arise from the synovium of joints, bursae, and tendon sheaths. Based on growth patterns and clinical behavior, TGCTs are classified into two subtypes: localized and diffuse (5-7). It is caused by a mutation in the stromal cells of the synovial membrane, resulting in overproduction of colony-stimulating factor 1 (CSF1). This overproduction lead to attracts of CSF1 receptors-expressing cells from the mononuclear phagocyte lineage, which constitute the bulk of the tumor mass (6, 8). It commonly affects the appendicular skeleton and only occasionally involves the axial skeleton(9). Diffuse type TGCT occur most frequently in the knee (64%), followed by the ankle (14%), hip (10%), feet (5%), and shoulder (1%) (10) . 

 TGCT shows variable signals on MRI, appearing hypointense to isointense on T1WI and heterogeneous hyperintense on T2WI. TGCT-D and has more hemosiderin deposition than localized (TGCT-L), resulting in low signal intensity on both T1WI and T2WI (11).

 TGCT-D lesions are metabolically active, osteolytic lesions with a sclerotic rim and prove intense radioactive glucose tracer (FDG) on PET scans, with reported maximum standardized uptake values (SUV_max_). These radiological and metabolic features can closely mimic metastatic malignancy making differentiation from secondary cancerous like D-TGCT particularly challenging (9).

 In this case, we describe a patient with complex history of OTC and unexpected discovery of TGCT-D. This rare tumor involved the entire left foot and ankle joint and initially mimicked osseous metastasis on ^18^F-FDG PET/CT. Notably. ^131^I whole-body scan does not show this lesion, underscoring the diagnostic challenges and the importance of multimodality imaging, histopathology, and clinical correlation in complex conditions. 

## Case presentation 

 We present the case of a 45-year-old man who was initially evaluated eight years prior for a progressively enlarging anterior neck mass. Neck ultrasonography at that time proven suspicious features concerning for malignancy, prompting fine-needle aspiration followed by total thyroidectomy. Histopathological examination confirmed the diagnosis of OTC. No adjuvant therapy was administered following the initial surgery, and the patient was subsequently lost to regular follow-up.

 Approximately seven years prior to presentation at our institution, the patient developed gradual swelling of the left ankle, associated with mild pain only when wearing tight-fitting shoes. As symptoms were minimal, no further diagnostic evaluation or treatment was pursued, and the condition remained clinically stable for several years.

 One year prior to the current presentation, the patient represented with recurrent neck swelling. Laboratory evaluation revealed an elevated non-stimulated serum thyroglobulin level of 60.1 µg/mL. Neck ultrasonography demonstrated multiple small hypoechoic nodules within both thyroid bed, suspicious for recurrent disease. Contrast-enhanced computed tomography of the neck confirmed these findings, further raising concern for disease recurrence. Surgical excision of the neck lesions was performed, and histopathology confirmed recurrent oncocytic thyroid carcinoma.

 Following surgery, the patient received radioactive iodine (RAI) therapy with a dose of 125 mCi. Post-therapy whole-body scintigraphy demonstrated radioiodine avid foci within the thyroid bed, consistent with residual or recurrent thyroid tissue. At that time, the stimulated thyroglobulin level was 107 µg/mL. 

**Figure 1:**
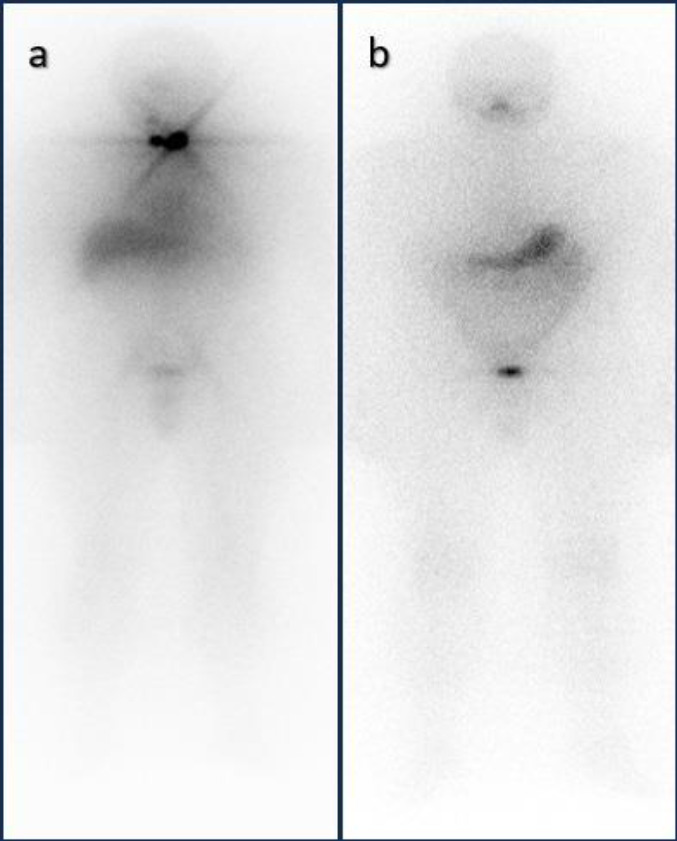
Whole body scan (WBS): **a**. post-therapy scan revealed residual thyroid tissue, **b**. diagnostic WBS yielded negative results

 However, at six-month follow-up, a diagnostic whole-body scan revealed a non–RAI-avid lesion in the right thyroid bed (Figure 1), accompanied by a further rise in serum thyroglobulin to 157 µg/mL, suggesting RAI-refractory disease.

**Figure 2:**
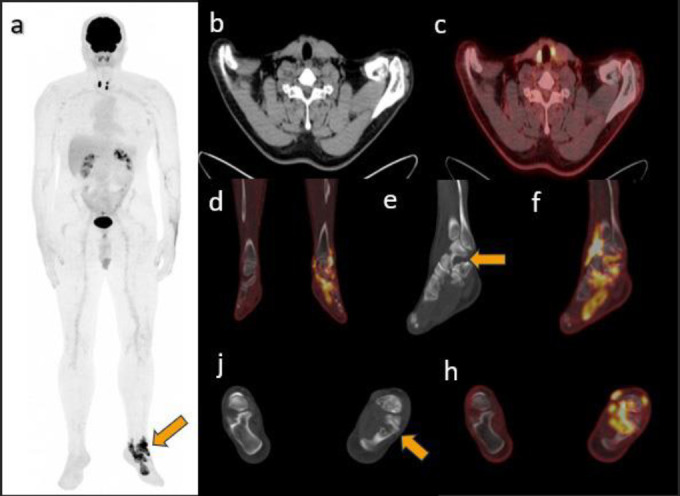
**a**. Maximum imaging projection (MIP), **b-c**. CT and fused images showing intense FDG uptake in thyroid bed, and **d-h**. Infiltrative soft tissue lesions involving left ankle and ventral surface of the foot with multiple lytic bone lesions (**thick arrow**)

 Following multidisciplinary discussion, ^18^F-FDG PET/CT was performed to evaluate for tumor dedifferentiation. The scan revealed three intensely FDG-avid soft-tissue lesions: two in the left thyroid bed (SUV_max_ up to 32.4) and one in the right thyroid bed (SUV_max_ up to 47.7). In addition, markedly hypermetabolic infiltrative soft-tissue lesions were identified involving the left ankle and plantar aspect of the foot (SUV_max_ up to 15.1), associated with multiple osteolytic lesions and cortical destruction of the calcaneus and several tarsal bones (Figure 2). These findings were initially interpreted as consistent with metastatic disease.

 Subsequent magnetic resonance imaging of the left foot demonstrated a large infiltrative soft-tissue mass extending from the distal pretibial region to the dorsal and plantar midfoot, measuring approximately 15.5×7×10 cm (anteroposterior × transverse × craniocaudal).

**Figure 3:**
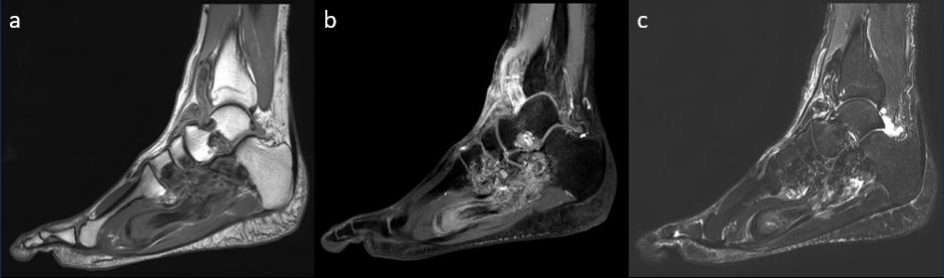
Left foot Magnetic Resonance Image (MRI) with contrast: shows an infiltrative soft tissue mass extending from distal pre-tibial level to the dorsal and plantar aspects of the midfoot showing extensive multifocal soft tissue and intra-articular involvement with tendinous, vascular encasement and causing multifocal bony erosions measuring roughly 15.5×7×10 cm (AP×TL×CC), **a.** It shows intermediate to hypointense signal on T1 weighted images, particularly peripheral low signal intensity, **b.** There is similar intermediate to hypointense signal on T2-weighted images with foci of hyperintense component, **c.** It shows heterogeneous post-contrast enhancement

 The lesion showed extensive intra-articular and periarticular involvement, encasement of adjacent tendons and vascular structures, multifocal bony erosions, and heterogeneous post-contrast enhancement (Figure 3).

 Ultrasound-guided biopsy of the left foot lesion revealed histopathological features consistent with (TGCT-D). This unexpected diagnosis explained the aggressive imaging appearance and clarified that the foot lesion was unrelated to metastatic OTC.

 Following multidisciplinary review, orthopedic consultation determined that surgical intervention was not indicated due to limited symptoms, with surgery to be reconsidered if functional impairment develops. For the recurrent RAI-refractory oncocytic thyroid carcinoma, external beam radiotherapy was initiated, and the patient remains under close follow-up.

## Discussion

 TSGT-D is a locally aggressive neoplasm that affects the appendicular skeleton, In the present case, the aggressive lesion of the left foot and ankle was initially suspected to represent metastatic disease from oncocytic thyroid carcinoma because of its known metastatic potential and intense FDG uptake(1). A similar diagnostic pitfall was reported by Kyoung Jin Chang et al., who described a vertebral FDG-avid lesion in a patient with papillary thyroid carcinoma that was diagnosed as TSGT-D, (9), mirroring our findings.

 These findings indicate that although FDG PET/CT is sensitive for detecting TGCT-D, its specificity in such cases is limited, and may lead to misinterpretation as malignancy. This is especially true in our case, where the known aggressiveness of OTC contributed to a false-positive result.(12, 13). Notably, no established SUV threshold currently exists to reliably distinguish benign from malignant TGCT.

 In patients undergoing whole-body ^18^F-FDG PET/CT for the evaluation of neoplastic diseases, TGCT-D may coexist with other malignancies, thereby posing significant diagnostic challenges. Al-Ibraheem A et al. reported a similar scenario in a patient with Hodgkin lymphoma, who demonstrated a complete treatment response except for a persistent focal FDG-avid lesion in the left knee. Biopsy confirmed this lesion to be TGCT, underscoring the importance of considering alternative diagnoses and incorporating MRI correlation in cases of diagnostic uncertainty (14).

 TGCT is generally not associated with RAI activity, as observed in our case and in the report by Loharkar et al. However, Cañete Sánchez et al.  described a rare case demonstrating RAI avidity in the wrist, which was interpreted as recurrent TGCT (15). The key point here is that TGCT is rarely detected on a ^131^I whole-body scan, in contrast to FDG PET/CT, which demonstrates high sensitivity for TGCT. This highlights the importance of using FDG PET/CT to ensure correct diagnosis and management, as proven this case.

 Multiple studies have reported intense FDG uptake in TGCT, consistent with our findings where the SUV_max_ was 15.1. In a retrospective observational study by Kohei Mizuta et al., involving 20 patients with TGCT, the mean SUV_max_ was 12.0±6.50. Comparable SUV_max_ values were also reported by Chun-Chieh Wu et al. and Chen et al. further supporting the reproducibility of FDG uptake in TGCT across different studies (12, 16, 17).

 To our knowledge, this is the first reported case to demonstrating the concurrent presence of two rare neoplasms, OTC and TGCT-D, identified on FDG PET/CT imaging. Associations between TGCT and other primary malignancies have been described, including papillary thyroid carcinoma, neurofibromatosis, choroidal melanoma, chondroid metaplasia, and Hodgkin lymphoma (13, 14, 18, 19). 

 However, the underlying relationship between TGCT and these malignancies remains unclear and no direct molecular, genetic, or signaling pathway overlap between OTC and

TGCT has been identified to date.

 Overall, this case underscores a critical clinical message: in patients with known malignancy, FDG-avid musculoskeletal lesions should not be automatically assumed to represent metastatic disease. Careful integration of clinical context, cross-sectional imaging, particularly MRI, and histopathological confirmation remains essential to avoid misdiagnosis and to ensure appropriate patient management.

## Conclusion

 This case highlights the diagnostic challenge posed by FDG-avid lesions in patients with oncocytic thyroid carcinoma, where TGCT-D may mimic metastatic disease on ^18^F-FDG PET/CT. Although PET/CT was useful in detecting metabolically active disease, its limited specificity underscores the need for careful clinical correlation and histopatho-logical confirmation. To our knowledge, this is the first reported coexistence of TGCT-D and OTC identified on FDG PET/CT imaging, adding to the growing list of malignancies associated with TGCT-D. Further research is needed to clarify these relationships and improve differentiation between benign and malignant lesions in imaging studies. 

## References

[1] Syed A, Vanka SA, Escudero I, Ismail R, Krayem H (2022). Oncocytic cell carcinoma of the thyroid: a case report and an overview of the diagnosis, treatment modalities, and prognosis. Cureus.

[2] Sciubba DM, Petteys RJ, Kang S, Than KD, Gokaslan ZL, Gallia GL (2010). Solitary spinal metastasis of Hürthle cell thyroid carcinoma. Journal of Clinical Neuroscience.

[3] Rahbari R, Zhang L, Kebebew E (2010). Thyroid cancer gender disparity. Future oncology.

[4] Zhang K, Wang X, Wei T, Li Z, Zhu J, Chen YW (2024). Radioactive iodine therapy improves overall survival outcome in oncocytic carcinoma of the thyroid by reducing death risks from noncancer causes: A competing risk analysis of 4641 patients. Head & Neck.

[5] Stacchiotti S, Dürr HR, Schaefer IM, Woertler K, Haas R, Trama A (2023). Best clinical management of tenosynovial giant cell tumour (TGCT): A consensus paper from the community of experts. Cancer treatment reviews.

[6] Kager M, Kager R, Fałek P, Fałek A, Szczypiór G, Niemunis-Sawicka J (2022). Tenosynovial giant cell tumor. Folia Medica Cracoviensia.

[7] Ehrenstein V, Andersen SL, Qazi I, Sankar N, Pedersen AB, Sikorski R (2017). Tenosynovial Giant Cell Tumor: Incidence, Prevalence, Patient Characteristics, and Recurrence A Registry-based Cohort Study in Denmark. The Journal of Rheumatology.

[8] Palmerini E, Staals EL (2022). Treatment updates on tenosynovial giant cell tumor. Current Opinion in Oncology.

[9] Chang KJ, Byun BH, Moon HS, Park J, Koh JS, Kim BI (2014). Tenosynovial Giant Cell Tumor of Diffuse Type Mimicking Bony Metastasis Detected on F-18 FDG PET/CT. Nuclear Medicine and Molecular Imaging.

[10] Siegel M, Bode L, Südkamp N, Kühle J, Zwingmann J, Schmal H (2021). Treatment, recurrence rates and follow-up of Tenosynovial Giant Cell Tumor (TGCT) of the foot and ankle-A systematic review and meta-analysis. PLOS ONE.

[11] Choi WS, Lee SK, Kim JY, Kim Y (2024). Diffuse-Type Tenosynovial Giant Cell Tumor: What Are the Important Findings on the Initial and Follow-Up MRI?. Cancers (Basel).

[12] Chen EL, de Castro CM, Hendzel KD, Iwaz S, Kim MA, Valeshabad AK (2019). Histologically benign metastasizing tenosynovial giant cell tumor mimicking metastatic malignancy: A case report and review of literature. Radiology Case Reports.

[13] Takeuchi A, Yamamoto N, Hayashi K, Miwa S, Takahira M, Fukui K (2016). Tenosynovial giant cell tumors in unusual locations detected by positron emission tomography imaging confused with malignant tumors: report of two cases. BMC Musculoskeletal Disorders.

[14] Al-Ibraheem A, Moghrabi S, Abdlkadir AS, Haidar M, Jaber O (2024). Unmasking Coincident Hodgkin Lymphoma and Giant Cell Tumor: Insights from [(18)F] FDG PET/CT. Asia Oceania Journal of Nuclear Medicine and Biology.

[15] Cañete Sánchez FM, Romero Robles LG, Boulvard Chollet XL, Mangas Losada M, Garrastachu P, Cabrera Villegas A (2022). Unusual Uptake of [(131)I] in a Tenosynovial Giant Cell Tumour Relapse in a Patient with Differentiated Thyroid Cancer. Molecular Imaging and Radionuclide Therapy.

[16] Mizuta K, Oshiro H, Tsuha Y, Tome Y, Nishida K (2023). Imaging characteristics of tenosynovial giant cell tumors on 18F-fluorodeoxyglucose positron emission tomography/computed tomography: a retrospective observational study. BMC Musculoskeletal Disorders.

[17] Wu C-C, Chou Y-H, Yang W-J, Hsu J-D, Tseng H-C, Lee Y-C (2019). Recurrent diffuse-type tenosynovial giant cell tumor of the left ankle: a case report. Therapeutic Radiology and Oncology.

[18] Loharkar S, Basu S (2023). Tenosynovial Giant Cell Tumor of Foot in a Patient with Radioiodine-Refractory Thyroid Cancer: Imaging Findings on FDG-PET/CT, MRI, and Radioiodine Scan. World Journal of Nuclear Medicine.

[19] Pina S, Fernandez M, Maya S, Garcia RA, Noor A, Pawha PS (2014). Recurrent Temporal Bone Tenosynovial Giant Cell Tumor with Chondroid Metaplasia: The Use of Imaging to Assess Recurrence. The Neuroradiology Journal.

